# Neuropsychiatric symptoms and metamemory across the life span: psychometric properties of the German Multifactorial Memory Questionnaire (MMQ)

**DOI:** 10.1007/s00415-024-12402-4

**Published:** 2024-05-08

**Authors:** Sophia Rekers, Josephine Heine, Angelika I. T. Thöne-Otto, Carsten Finke

**Affiliations:** 1 Department of Neurology, Universitätsmedizin Berlin, Freie Universität Berlin and Humboldt-Universität zu Berlin, Berlin, Germany; 2 Berlin School of Mind and Brain, Humboldt-Universität zu Berlin, Berlin, Germany; 3 Department of Psychology, Humboldt-Universität zu Berlin, Berlin, Germany; 4 Clinic for Cognitive Neurology, University of Leipzig, Max-Planck-Institute of Human Cognitive and Brain Sciences, Leipzig, Germany

**Keywords:** Multifactorial Memory Questionnaire, MMQ, German normative data, Metamemory, Subjective memory impairment, Subjective cognitive decline

## Abstract

**Objective:**

We assessed the psychometric properties, established normative data for the German Multifactorial Memory Questionnaire (MMQ), and analyzed its association with neuropsychiatric factors across the life span to provide a validated metamemory assessment for a German-speaking population.

**Methods:**

The three MMQ scales (memory satisfaction, self-rated ability, and strategy application) were translated into German, considering cultural, linguistic, and conceptual aspects. To validate the MMQ and assess associations with neuropsychiatric factors, the Complainer Profile Identification, Geriatric Depression Scale, Beck Anxiety Inventory, Pittsburgh Sleep Quality Index, and Short-Form-Health Survey were applied in an online study in 336 healthy participants with follow-up after 8 months.

**Results:**

Psychometric evaluation of the German MMQ showed normal distribution of all scales and good to excellent validity, internal consistency, and retest reliability. We provide percentiles and normative data for *z*-score conversion. Importantly, even subclinically elevated scores in depressiveness and anxiety were associated with decreased memory satisfaction and self-rated ability. Furthermore, although the influence of age on the German MMQ scales was minimal, effects of neuropsychiatric factors such as sleep quality, anxiety, and depressiveness on MMQ *Satisfaction* and *Ability* varied across the life span.

**Conclusions:**

Our study provides a validated German translation of the MMQ with normative data and reliability measures, including reliable change scores. We show the impact of neuropsychiatric factors on the MMQ scales across the life span and emphasize the relevance of a multifactorial approach to metamemory as a measure of individualized everyday functionality and the importance of including neuropsychiatric factors into both research and clinical assessments of metamemory.

**Supplementary Information:**

The online version contains supplementary material available at 10.1007/s00415-024-12402-4.

## Introduction

Metamemory refers to the ability to monitor or make judgments about one’s own memory processes [1–3] and is neutral in its valence about the subjective judgment. In contrast, subjective memory impairment (SMI) or complaints specifically refer to a non-functional state of memory, and subjective cognitive decline (SCD) describes self-perceived worsening of cognition in general or memory specifically. Although these terms are frequently used interchangeably, distinctive elements of SMI have been associated with the affective component of worries or satisfaction and impact on everyday life [4, 5].

SMI is frequently reported in many neurological disorders, e.g., epilepsy [6], Parkinson’s disease [7], or multiple sclerosis [8], but can also increase with age [9]. In memory clinics, clinicians face the challenge that sometimes memory complaints cannot be objectified by standard neuropsychological assessments, but still precede future cognitive decline. Indeed, research from large populations [10] and individuals with increased biomarker-based risk for Alzheimer’s disease [11, 12] indicates that subjective memory decline is associated with an increased risk for conversion to mild cognitive impairment and dementia even in cognitively unimpaired people [5, 13]. Furthermore, SMIs are associated with an Alzheimer’s disease-like gray matter atrophy pattern [14] and medial temporal lobe volume loss [11, 15]. Beside this potential for identifying cognitive decline earlier than standardized cognitive tests, SMI can also be a more individualized approach to assessing everyday impairment, especially in high-performing individuals lacking baseline assessments [16, 17].

To operationalize subjective memory, existing approaches vary regarding covered domains and time frames, administration modes, and number and phrasing of items and answer scales [4]. Common methods include asking for a judgment of the extent of memory decline, e.g., Memory Complaint Questionnaire [18], memory complaint frequency, like in the Complainer Profile Identification [19], or rating how often memory-related tasks present a problem, e.g., Memory Functioning Questionnaire [20]. Some approaches also integrate strategy use and external judgment, such as the Subjective Memory Complaints scale [21]. Lastly, one-item binary assessments of the presence or absence of subjective complaints are frequently applied, but might lack sensitivity to identify people with high-risk profiles. In contrast, continuous measures enable evaluating metamemory changes over time and investigating associations between subjective judgments and other outcomes. However, many questionnaires lack psychometric assessment, normative data, and appropriate cultural adjustments [22] and approach metamemory as a singular factor.

The Multifactorial Memory Questionnaire—MMQ [17] offers a multifactorial approach to SMI or metamemory. It dissociates the scales *Satisfaction*, *Ability*, and *Strategy,* which allows to take differential confounding factors into account. *Dissatisfaction*, concerns, or worries about one’s own memory performance, are important predictors for the development of symptomatic Alzheimer’s disease [5, 10] and conversion to objective cognitive impairment [23], but are also associated with affective disorders like depression [24] and confounded by depressiveness [25]. In contrast, self-rated *ability* directly relates to everyday memory function, but is impacted by monitoring ability. Skewed judgments may result from underlying brain pathologies or be biased by the degree of confrontation and self-reflection. Lastly, mnemonic *strategies* have a complex relationship to subjective memory ability and satisfaction. While they are frequently applied by high-performing individuals and trained in cognitive interventions [26–28], an increased use of everyday memory strategies is consistently associated with more memory complaints [29–31]. This highlights the potential confounding effect of strategy items in unifactorial questionnaires. Therefore, the additional application time of a multifactorial questionnaire differentiating memory satisfaction, subjective performance, and strategies use can bring significant value by ensuring accurate assessment and facilitating effective treatment planning.

Here, we present the German translation and normative data of the Multifactorial Memory Questionnaire (MMQ [17]) and (i) provide a culturally and linguistically appropriate transfer, (ii) assess its psychometric properties, and (iii) investigate the differential influence of neuropsychiatric factors including depressiveness, anxiety, sleep, and health-related quality of life on its scales *Satisfaction*, *Ability*, and *Strategy*. Multifactorial metamemory questionnaires have numerous potential applications in research and clinical contexts, including monitoring of longitudinal changes, and evaluating the efficacy and contributing factors of rehabilitation and training interventions. Additionally, they can function as a standardized assessment of self-efficacy and compensatory mechanisms. However, since depressiveness, anxiety, physical and mental health, and sleep differentially affect memory across the life span [29, 32, 33], neuropsychiatric symptoms need to be considered when evaluating metamemory.

## Materials and methods

### Participants and data acquisition

**Fig. 1:**
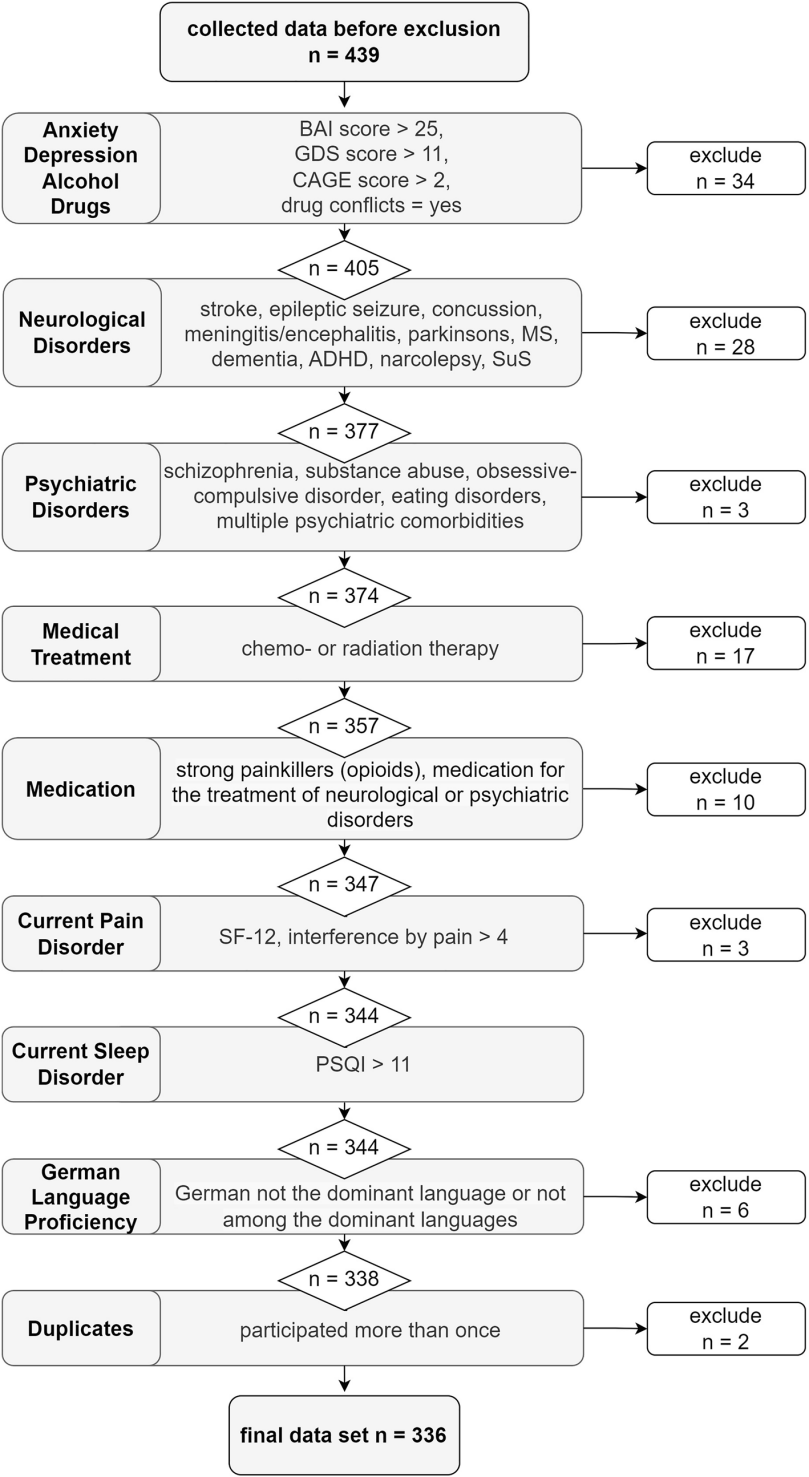
Sample flowchart with exclusion criteria

We recruited 439 healthy participants between April 2020 and April 2021 using the online tool SoSci Survey [34]. Participants were invited through the hospital-based website, community clubs and organizations for senior citizens, and social media posts. Inclusion criteria were age ≥ 18 years and adequate German language proficiency. Exclusion criteria were a history of neurological or psychiatric disorders, clinically relevant screening scores for mood, pain, and sleep disorders, substance abuse, as well as previous chemo- or radiation therapy or current medication suspected to interfere with cognition (for a detailed flowchart of the exclusion process of participants please see Fig. 1). Of the 439 individuals who participated, 336 participants fulfilled the predetermined inclusion criteria for the baseline assessment. Demographic information on the final sample is presented in Table 1 and Fig. 6 illustrates the age and gender distribution of the norm sample. For the retest assessment, included participants were invited to participate again on average 8 months later (*SD* = 1.27 months), mirroring the interval of biannual or annual clinical visits. A total of 122 participants responded and 94 participants were included who could be matched to their baseline assessment based on their participation ID.

**Table 1:** Demographic information and clinical self-assessment scales

	Mean	SD	Range
Age (years)	51.18	±17.63	19–86
Geriatric Depression Scale (GDS)	1.84	±2.34	0–11
Beck Anxiety Inventory (BAI)	5.47	±5.12	0–25
Physical quality of life (SF-12)	39.99	±3.47	20.73–48.44
Mental quality of life (SF-12)	44.54	±5.62	22.79–53.61
Complainer Profile Identification (CPI)	1.96	±0.56	1–4
CPI attention	1.88	±0.61	1–4
CPI executive	2.01	±0.64	1–4.33
CPI memory	1.93	±0.61	1–4

	Category	*n*	Share of the sample (%)
Gender	Female	231	69
	Male	105	31
Education	High school (12–13 years)	203	60
	Upper secondary (11–12 years)	35	10
	Secondary (10 years)	89	26
	Lower secondary (8–9 years)	9	3
Current work status	Full-time	175	52
	Part-time	43	13
	Unemployed	1	<1
	Retired	97	29
	Other	20	6
Household income^a^	<1.000€	11	3
	1.000–<2.000€	90	27
	2.000–<3.000€	123	23
	3.000–<4.000€	54	16
	>4.000€	58	17

^a^Monthly net income

To ensure data fidelity of the online study, we incorporated rigorous compliance measures in the study design and quality checks during data processing. These included the contextualization of the data collection as normative data for clinical evaluation, thorough physical and mental health-related questionnaires, and previous warnings that these questionnaires are included, contact details of services who provide specific help and information in situations of psychological distress, optional possibility to receive feedback on questionnaire scores with clinical relevance, possibility to self-declare untrue data, and double participation checks.

### Translation process into German

Instructions, items, and rating scales were translated in an iterative approach considering linguistic and cultural conventions in German-speaking countries and the precision of the underlying constructs. To ensure this, the first transfer from English to German was done by two native German speakers (JH, SR) with high English proficiency. The resulting translation was proofread by a native English speaker with high German proficiency (Graham Cooper) and edited. This version was checked regarding grammar and spelling by a German linguist (Julia Heine). After integrating these revisions, the second version was created. To ensure the precision of the underlying constructs, we then chose an automated back-translation of this second version into English using the AI-based software “DeepL”. The authors of the original MMQ scales (Angela Troyer, Jill Rich) checked this automated translation and their edits were integrated to create the final version of the German MMQ. For details on each item, see Supplementary information 1.

### Questionnaires

The Multifactorial Memory Questionnaire (MMQ) developed by Troyer & Rich [17] includes three scales: (1) *Satisfaction*, encompassing 18 statements on the subjective overall appraisal of and worries associated with one’s memory; (2) *Ability*, which assesses the perception of one’s own day-to-day memory performance and includes 20 common memory mistakes that are rated regarding their frequency in the past 2 weeks; and (3) *Strategy*, comprising 19 practical memory strategies and aids which are rated analogously in terms of their frequency. Shaikh et al. [35] found that factor analyses supported that the latter scale can be further divided into internal (i.e., mental) and external (i.e., using auxiliary elements in the environment) memory strategies. Each MMQ item is scored on a 5-point Likert scale from 0 to 4, after recoding inverted items. The resulting score for each scale is interpreted separately with low scores indicating low satisfaction, low self-rated ability (mistakes occur frequently), or infrequent use of strategies. The MMQ and its translation are freely available for researchers and clinicians for non-commercial use at the Baycrest website (https://www.baycrest.org/mmq).

To validate the German MMQ, we also applied the questionnaire Complainer Profile Identification (CPI), which assesses subjective complaints in the domains memory, attention, and executive functions [19]. It consists of 17 items describing cognitive complaints, which are rated on a 5-point Likert scale regarding their frequency (“never” to “very often”). Affective symptoms were assessed using the Geriatric Depression Scale (GDS-15) [36, 37] for depressive symptoms and the Beck Anxiety Inventory (BAI) [38, 39] for symptoms of anxiety. Furthermore, we assessed sleep quality using the Pittsburgh Sleep Quality Index components subjective sleep quality, use of sleeping medication, and daytime dysfunction (PSQI [40, 41]) and mental and physical health-related quality of life using the Short-Form-Health Survey (SF12) [42, 43]. Moreover, we acquired demographic information, including age, gender, language proficiency, medication, smoking habits, and potential alcohol and drug abuse (see Table 1 and Fig. 1).

### Statistical analysis

Preprocessing and all analyses were performed using R, Version 3.6.1 [44]. Details on used packages and versions are available in the analysis script, which can be accessed at the Open Science Framework (https://osf.io/x6e8f). The level of statistical significance for all analyses was set at *p* < 0.05. The psychometric properties of the German MMQ scales were investigated regarding variability (distribution, skewness, and excess kurtosis), convergent validity assessed by correlations with the CPI scales on an individual level and considering intercorrelations using multiple regression analyses. Furthermore, we tested the MMQ scales’ internal consistency using Cronbach's alpha, retest reliability using Pearson’s product–moment correlation (*r*), and calculated reliable change scores for the MMQ scales by multiplying the standard error of the difference (SE_diff_), based on the standard errors of the measurement (SEM) at baseline and retest, by the *z*-score 1.96 (95% CI) [45, 46]. To assess correlates of the MMQ scales, we assessed their associations with demographic and neuropsychiatric measures using Pearson’s product–moment correlation for approximately normally distributed variables (skewness <|2| and excess kurtosis <|4|) [47]. When comparing two groups, *t* test for independent samples was used, when homogeneity of variance could be assumed based on Levene’s test. For regression models, significant predictors on an individual level were entered simultaneously. Successive predictor reduction for identification of the most parsimonious model was conducted by excluding non-significant predictors. The three age groups for the analyses across the life span were identified based on age tertiles in the sample to ensure approximately equal group sizes (young (18–40 years) *n* = 113, middle-aged (41–60 years) *n* = 112, older adults (61–86 years) *n* = 111).

## Results

### Psychometric properties

#### Variability

**Fig. 2:**
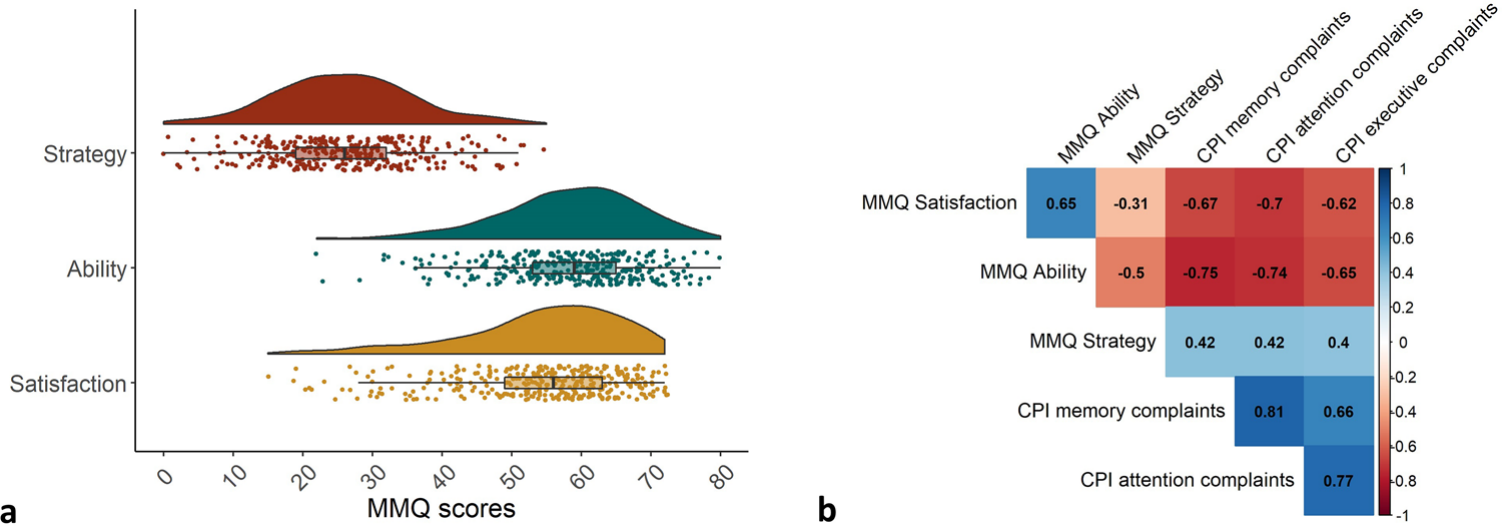
**a** MMQ variability: rain cloud plots illustrating the normal distribution of MMQ scales. **b** MMQ validity: construct validity correlation matrix of MMQ scales showing significant Pearson correlation coefficients with CPI scales

Prior to computing percentiles, the shape of the distribution for each scale was analyzed to test for normal distribution. All skewness and excess kurtosis values were well within the range of − 1.0 to 1.0 for the three MMQ scales, indicating normal distribution (*Satisfaction*: skewness = − 0.92, kurtosis = 0.55; *Ability*: skewness = − 0.59, kurtosis = 0.48; *Strategy*: skewness = 0.13, kurtosis = 0.05; Fig. 2A). Further details of the distribution of each MMQ scale, including mean and standard deviation for the calculation of standardized norm scores like *z-*scores, are provided in Table 2.

**Table 2:** Distribution and retest information of the MMQ scales in the German normative group

Baseline	*N*	Mean±SD	SE mean	Range
MMQ *Satisfaction*	336	54.26±11.67	0.64	15–72
MMQ *Ability*	336	58.21±10.04	0.55	22–80
MMQ *Strategy*	336	25.99±9.95	0.54	0–55
Internal	336	10.32±6.09	0.33	0–34
External	336	15.67±5.20	0.28	0–28

Retest *(8 months)*	*N*	Test–retest reliability [95% CI]	SE_diff_	Reliable change score^a^
MMQ *Satisfaction*	94	0.84 [0.76, 0.89]	6.96	13.64
MMQ *Ability*	94	0.81 [0.72, 0.87]	6.39	12.52
MMQ *Strategy*	94	0.76 [0.66, 0.83]	6.96	13.63

^a^Reliable Change Index (RCI) * 1.96 (*p* < 0.05)

#### Validity

To assess the validity of the German MMQ, we analyzed its associations with the scales memory, attention, and executive complaints of the CPI. As shown in Fig. 2B, less complaints in all CPI domains were strongly associated with a higher MMQ *Satisfaction* and self-rated *Ability* and a medium-sized decrease in use of strategies in the MMQ. Detailed test statistics on the correlations of the MMQ scales with questionnaire measures can be found in Table 6.

#### Reliability

We found excellent internal consistency for the MMQ scales *Satisfaction* (*α* = 0.93, 95% CI [0.92, 0.94]) and *Ability* (*α* = 0.90, 95% CI [0.89, 0.92]) and good internal consistency for the *Strategy* scale (*α* = 0.84, 95% CI [0.81, 0.86]). As reported in Table 2, we also found good test–retest reliability for the scales *Satisfaction* and *Ability* and acceptable test–retest reliability for the MMQ *Strategy* scale after an average interval of 8 months (range: 3.30–9.13, SD = 1.27). Table 2 also provides reliable change scores for each scale (95% CI), i.e., the number of points that indicate a clinically significant change on the respective scale.

### Normative data and correlates of metamemory

#### Demographic variables and questionnaire data

Age had a small, but significant association with MMQ *Satisfaction* (*r* =  − 0.11, 95% CI [− 0.21,0.00], *t*(334) =  − 1.97, *p* = 0.0499) and *Ability* (*r* =  − 0.13, 95% CI [− 0.24, − 0.03], *t*(334) =  − 2.45, *p* = 0.015), but not with *Strategy* (*t*(334) =  − 1. 44, *p* = 0.150). Men and women did not differ with respect to memory satisfaction (*t*(334) = 1.28, *p* = 0.201) or self-rated ability (*t*(334) = 0.98, *p* = 0.328), but men scored on average 2.41 points more on the *Strategy* scale than women (*d* =  0.24, *t*(334) =  − 2.07, *p* = 0.039). The level of education did not impact any of the three MMQ scales (*Satisfaction*: *χ*^*2*^(3) = 0.62, *p* = 0.892; *Ability*: *χ*^*2*^(3) = 0.50, *p* = 0.918; *Strategy*: *F*(3,332) = 0.90, *p* = 0.443). Considering the very small effect of age on *Satisfaction* and *Ability*, and the small effect of gender on *Strategy*, we decided to include all age groups into one normative sample in accordance with the original MMQ normative data by Troyer and Rich [17]. The normative data using percentile ranks for *Satisfaction* and *Ability* are presented in Table 3 and for the *Strategy* scale in Table 4 with recommendations for interpretation provided in Table 5.

**Table 3:**
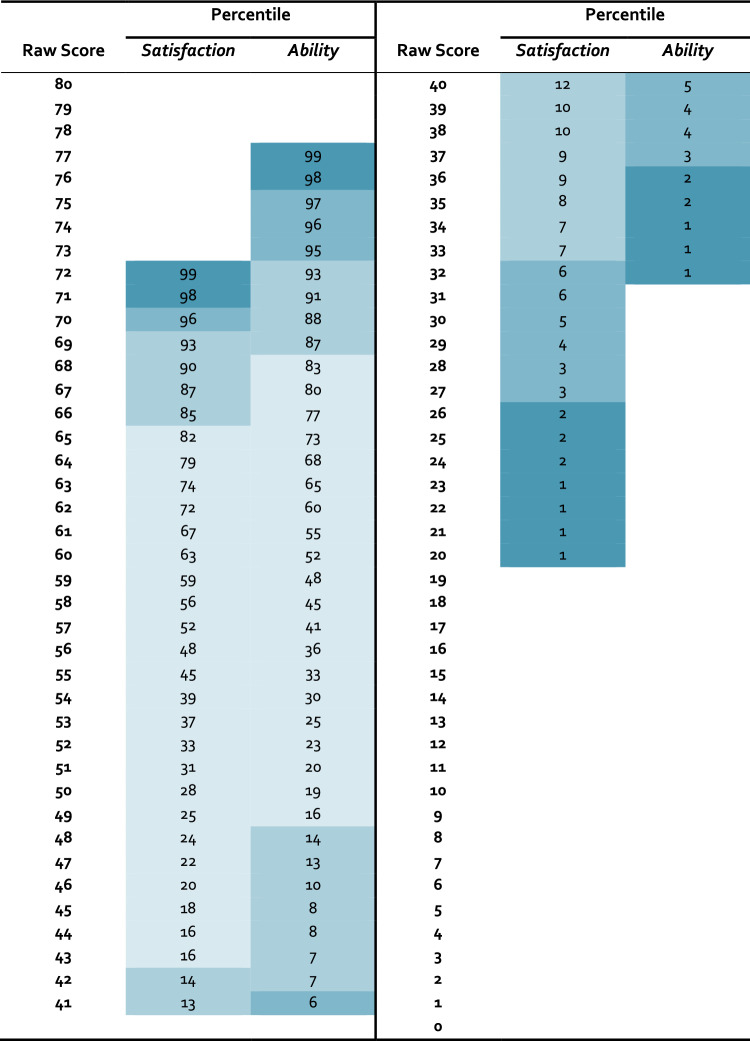
Percentiles for the MMQ scales *Satisfaction* and *Ability* (*N* = *336*)

**Table 4:**
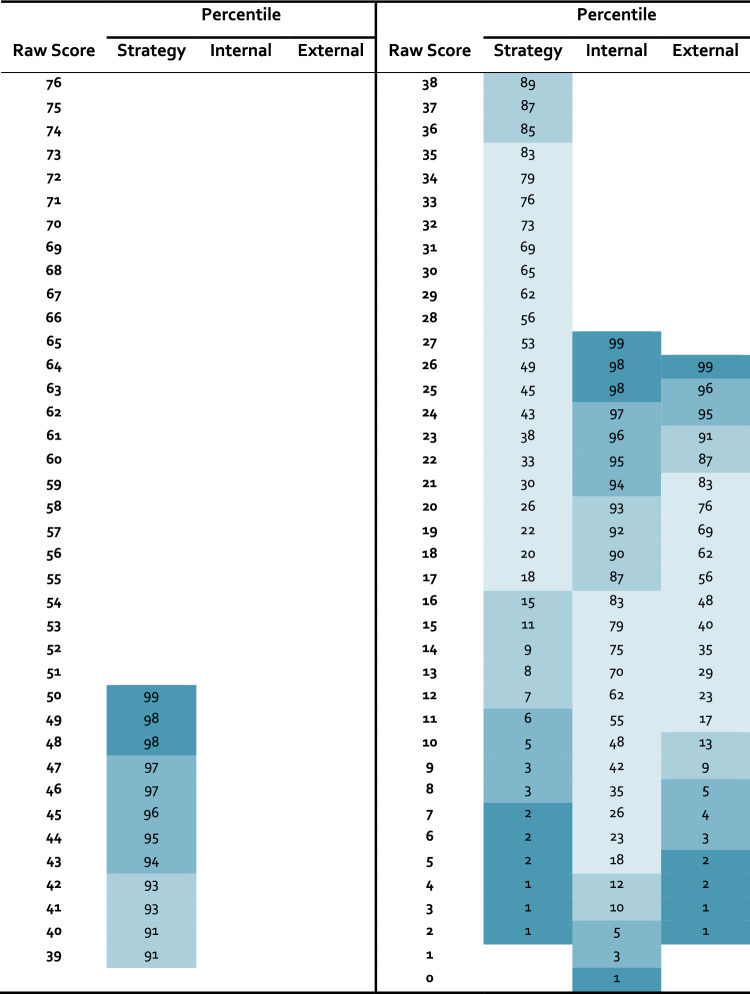
Percentiles for the MMQ Strategy scales (*N* = *336*)

**Fig. 3:**
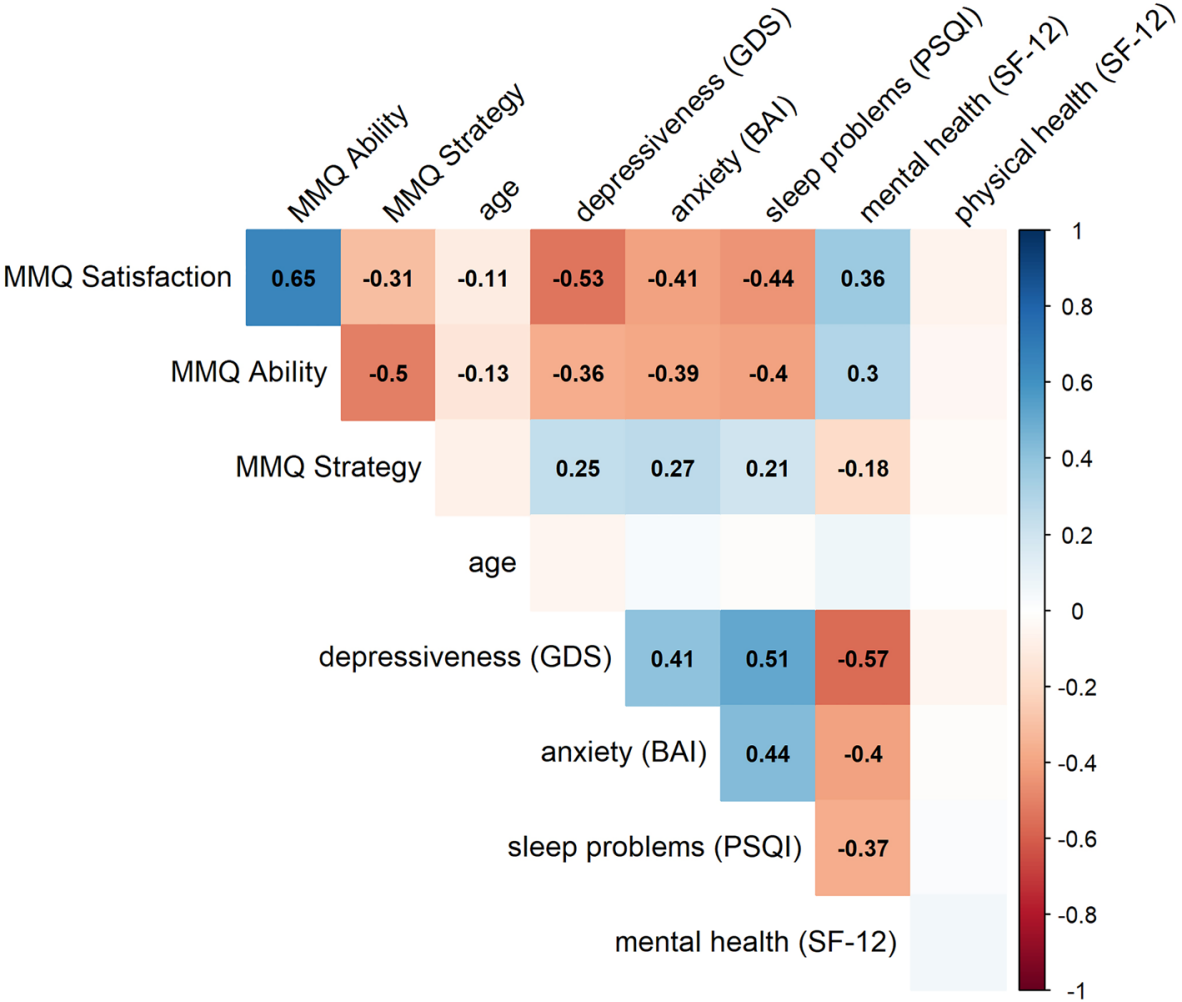
Correlation matrix of the MMQ scales with age and neuropsychiatric self-report measures. Pearson correlation coefficients are portrayed for significant correlations and show that MMQ *Satisfaction* and *Ability* scores decrease minimally with increased age and increase with lower depressiveness (GDS), anxiety (BAI), and sleep problems (PSQI), and better mental health (SF-12), with reversed pattern for S*trategy* scores

**Fig. 4:**
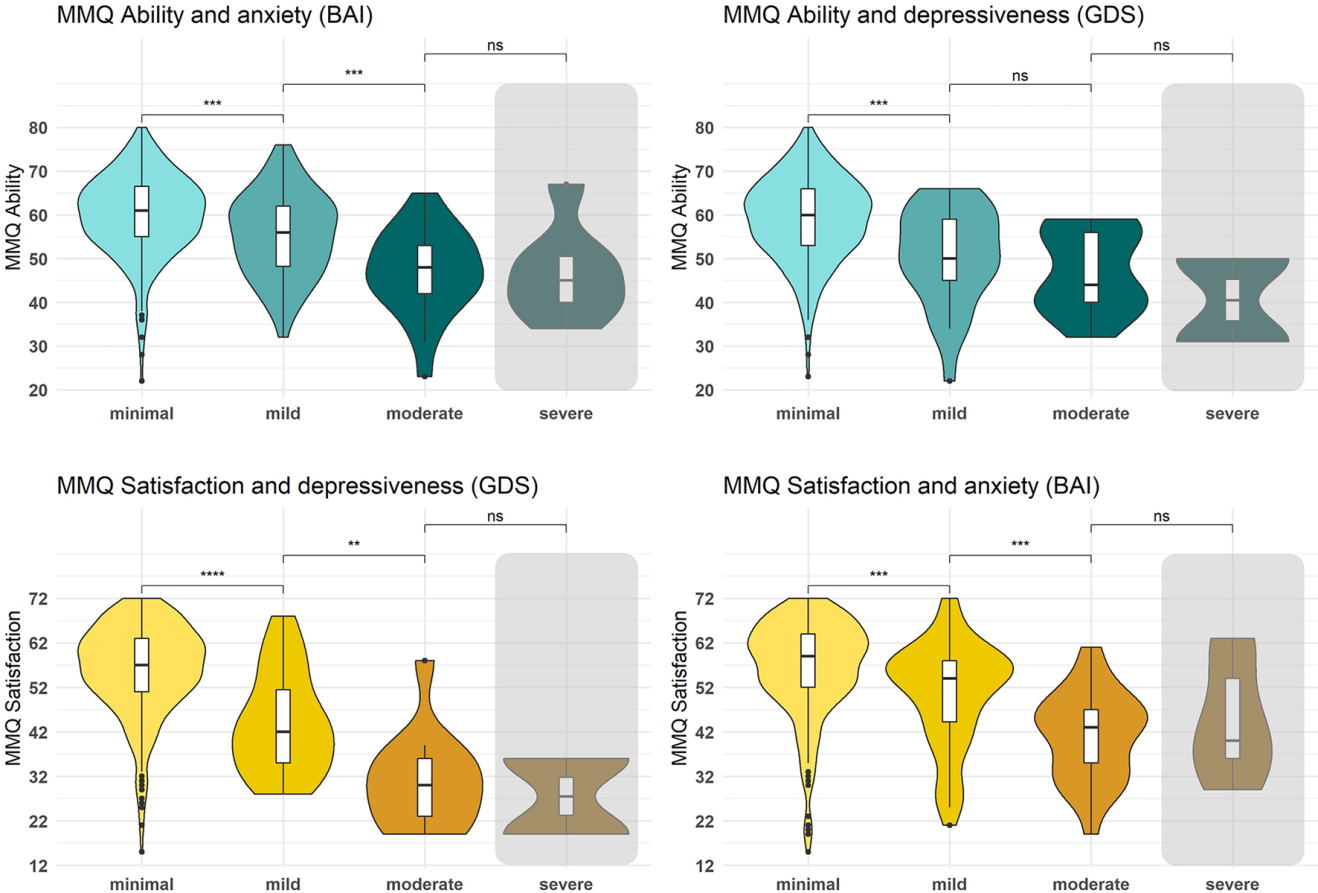
MMQ *Ability* and *Satisfaction* scores across levels of anxiety and depressiveness. Increases of anxiety and depressiveness levels from minimal to mild already significantly decrease self-rated ability and satisfaction. Participants with severe levels, who are excluded from other analyses, are portrayed by the gray shading, and do not differ significantly in their *Ability* and *Satisfaction* scores in comparison to participants with moderate anxiety and depressiveness

Based on the acquired questionnaire data (Fig. 3), we found that higher scores on MMQ scales *Satisfaction* and *Ability* were correlated with lower depressiveness (GDS), lower anxiety (BAI), less sleep problems (PSQI), and better mental health (SF-12), while the opposite pattern was observed for the *Strategy* scale. Physical health (SF-12) was not correlated with any of the collected measures. Detailed statistics on the correlations can be found in Table 6. Interestingly, the effect that higher anxiety and depressiveness are associated with lower *Satisfaction* and *Ability* was already seen at subclinical levels of anxiety (BAI) and depressiveness (GDS) with a gradual decrease of *Ability* and *Satisfaction* scores from minimal to mild and moderate anxiety and depressiveness (Fig. 4). Importantly, this is not specific to clinically relevant severe levels. In fact, even participants with mild compared to no or minimal anxiety or depressiveness show significantly lower subjective memory ability (BAI: *d* = 0.47, *t*(311) = 3.42, *p* = 0.001; GDS: *d* = 0.87, *t*(328) = 4.31,* p* < 0.001) and memory satisfaction (BAI: *d* = 0.51, *t*(311) = 3.70, *p* < 0.001; GDS: *d* = 1.15, *t*(328) = 5.75, *p* < 0.001).

#### Neuropsychiatric correlates of memory satisfaction and self-rated memory ability across the life span

**Fig. 5:**
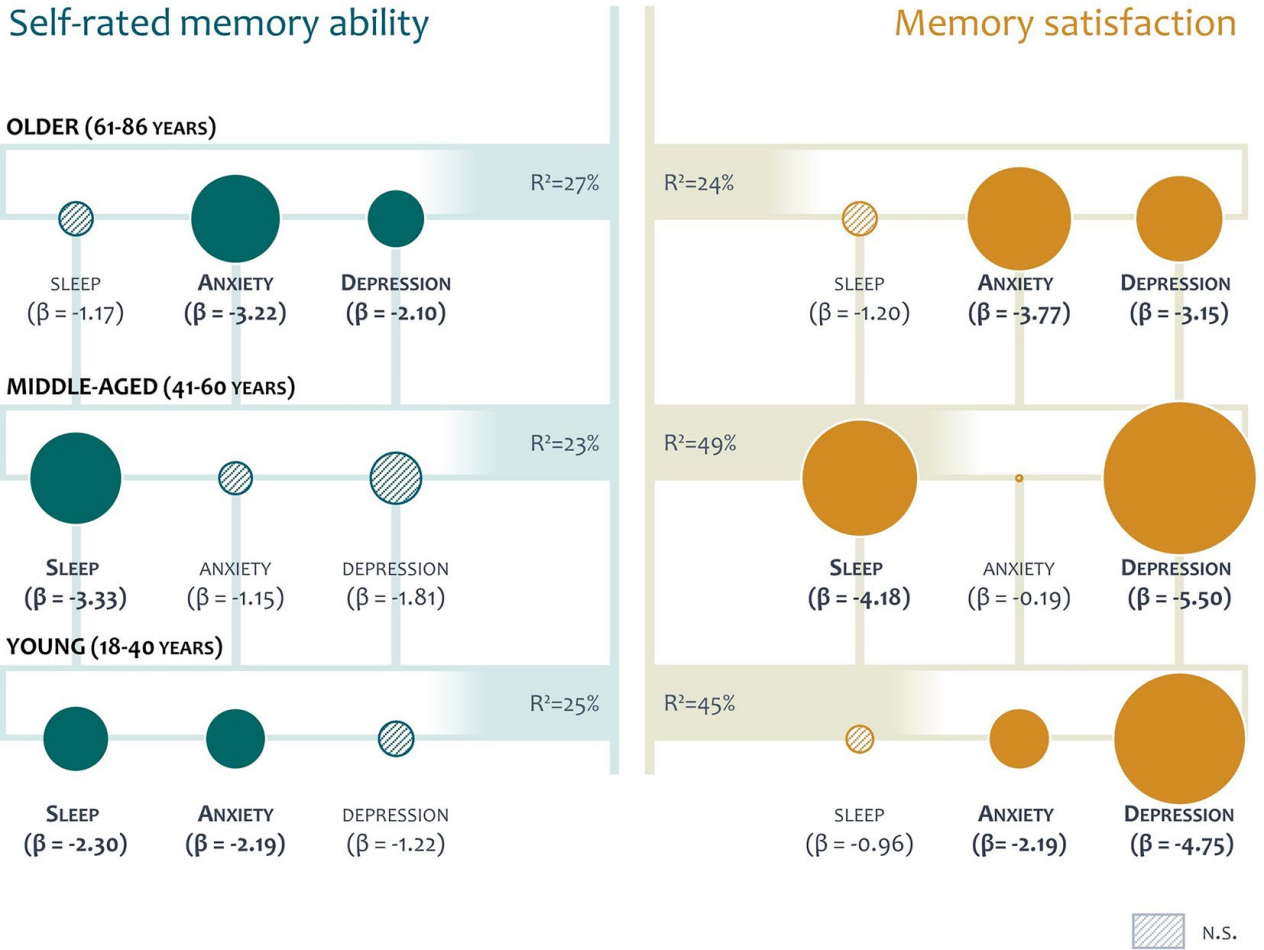
Predictors of self-rated memory ability and memory satisfaction across the life span. Regression models are shown for the three age groups with their explained variance (*R*^2^) using the predictors sleep problems (PSQI), anxiety (BAI), and depressiveness (GDS). *β* represents the predictors’ standardized regression weights, i.e., a change of one standard deviation on the predictor scale results in a change of *β* points on the MMQ scale. Depressiveness was associated with lower *Satisfaction* scores in all age groups and lower *Ability* scores in older adults. Anxiety affected both scales, but only in young and older adults, while sleep problems resulted in lower memory satisfaction in middle-aged adults and self-rated memory ability in young and middle-aged adults

**Fig. 6:**
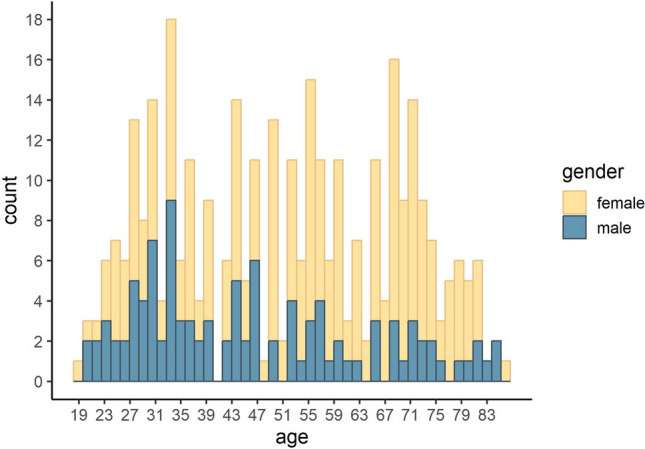
Histogram illustrating the age and gender distribution of the normative sample

Next, we calculated two regression models, using *Satisfaction* and *Ability* as the respective outcome measures, to account for the intercorrelation between age and the significant questionnaire measures. Here, we found that depressiveness, anxiety, and sleep problems remained significant predictors for memory satisfaction and self-rated ability, respectively, but mental health did not (*Satisfaction*: *t*(330) = 0.59, *p* = 0.556; *Ability*: *t*(330) = 1. 12, *p* = 0.263). Regression models using the significant predictors age, GDS score, BAI score, and PSQI score explained 36% of the variance in the *Satisfaction* score (*F*(4,331) = 47.32, *p* < 0.001) and 25% of the variance in the *Ability* score (*F*(4,331) = 27.97, p < 0.001). To address the question how these significant predictors affect the self-rated ability and memory satisfaction across the life span, we defined three age groups, i.e., young (18–40 years), middle-aged (41–60 years), and older adults (61–86 years) and calculated separate regression models for each age group with the MMQ scales *Ability* and *Satisfaction* as outcome measures (Fig. 5). Interestingly, we observed a differential impact of the three factors on metamemory, although they did not differ between age groups (depressiveness: *χ*^*2*^(2) = 2.16, *p* = 0.340; anxiety: *χ*^*2*^(2) = 3.00, *p* = 0.223; sleep: *χ*^*2*^(2) = 2.11, *p* = 0.349): Depressiveness was associated with poorer memory satisfaction in all age groups, but relevant for self-rated ability only in older adults. Anxiety affected both memory satisfaction and self-rated ability, but only in young and older adults. Lastly, sleep problems were associated with memory satisfaction in middle-aged adults and self-rated memory ability in young and middle-aged adults.

## Discussion

In this study, we assessed the German MMQ and its associations with neuropsychiatric factors in a healthy norm sample. First, we translated the original items and instructions considering cultural, linguistic, and conceptual aspects. Second, we assessed the psychometric properties and built normative data for the application of the MMQ in German speakers. The three scales *Satisfaction*, *Ability*, and *Strategy* exhibited a normal distribution, and we observed strong validity, internal consistency, and retest reliability in the German sample. In line with the original MMQ [17] and other translations of the MMQ [31], we found no or only small associations of age and gender with the MMQ scales. Furthermore, we found that neuropsychiatric factors such as anxiety, depressiveness, sleep problems, and mental, but not physical health, were associated with the German MMQ scores. Moreover, anxiety, depressiveness, and sleep problems differentially impacted memory satisfaction and self-rated ability across different age groups. Importantly, even subclinical levels of anxiety and depressiveness were associated with significantly reduced *Satisfaction* and *Ability* MMQ scores.

Concerning the psychometric properties of the German MMQ, the normal distribution of the three scales is advantageous for statistical analyses and enables converting raw scores not only to percentiles, but also to norm scores, like *z*-scores [48]. In addition, we confirmed the German MMQ’s convergent validity in its associations with the CPI, a German questionnaire on subjective cognitive complaints. *Satisfaction* and *Ability* showed large negative correlations, while *Strategy* demonstrated medium-to-large positive associations with the CPI scales memory, attention, and executive complaints. Our findings indicate that higher memory satisfaction and self-reported ability are linked to fewer cognitive complaints, whereas using more memory strategies is correlated with more cognitive complaints. Importantly, associations with the CPI attention and executive scales are reasonable considering the inclusion of some CPI memory items in the attention scale, as well as the overall high item-total correlations and internal consistency of the CPI total score (*α* = 0.87) [19]. Furthermore, several MMQ items require metamemory judgments about everyday memory tasks and prospective memory, where attention and executive functions are essential [49].

Regarding reliability, we found good (*Strategy*) to excellent (*Satisfaction* and *Ability*) internal consistency. Especially for clinicians, using the reliability to calculate confidence intervals is highly recommended (e.g., Crawford & Garthwaite, 2009). Furthermore, this finding supports the three-scale design for the German MMQ, although evidence suggests a potential division of the *Strategy* scale into internal and external strategies with factor analyses supporting both a 3- and 4-factor model of the MMQ [35, 50]. Thus, interpreting the *Strategy* scale as one or two scales is valid, depending on the user’s needs. We provide normative data for both interpretations. Moreover, we found acceptable (*Strategy*) to good (*Satisfaction* and *Ability*) retest reliability, even after an extended retest interval of 8 months. The derived reliable change scores can serve as valuable indicators of clinically relevant changes after an intervention period or during follow-up monitoring.

The multifactorial approach of the MMQ, which recognizes each scale as a separate factor, is a key advantage, allowing for separate interpretation of three dimensions of metamemory [17]. Although the MMQ scales *Satisfaction* and *Ability* are strongly associated, they assess different aspects of metamemory, measuring the affective appraisal of memory vs. self-rated frequency of memory mistakes. The finding that increased use of memory strategies correlates with lower self-rated ability and memory satisfaction is in line with MMQ studies in other languages [29, 31]. Although mnemonic strategies can be relevant in high-demand memory challenges and task-specific strategy use has been related to better performance [51], MMQ’s strategies are more applicable to everyday situations where healthy individuals typically do not require mnemonic techniques. Thus, an above average strategy use may indicate a subjective need for everyday functioning. However, the *Strategy* score can also assist to plan and monitor cognitive interventions in individuals who experience memory impairment and require compensatory strategy use for daily functioning [52].

In this study, we found minimal associations of the MMQ with demographic factors. Age had a small impact on *Satisfaction* and *Ability*, while men scored slightly higher on the *Strategy* scale than women, with no effect of education on any MMQ scale. Given the small effect sizes, we did not divide the norm tables by age or gender, considering the benefits of a larger norm sample. These findings on the small-to-negligible impact of demographic variables, although in contrast to some findings in metamemory in general [9], are in line with other MMQ translations [31, 53, 54].

However, it is important to consider participant age when examining the varying influence of neuropsychiatric factors. Our results show that even subclinical levels of anxiety and depressiveness affect *Satisfaction* and *Ability* scores and anxiety impacted young and older but not middle-aged adults, consistent with prior research on SMI and affective symptoms in elderly people [55, 56]. Moreover, depressiveness most strongly and robustly affected memory satisfaction, likely due to depression-associated worries, negative self-beliefs and aging stereotypes [19, 57], and strong overlap of some *Satisfaction* items with affective symptoms (e.g., MMQ Satisfaction: “I feel unhappy when I think about my memory ability.”). Anxiety might lower self-rated ability and memory satisfaction through increased uncertainty intolerance and health monitoring [58]. Sleep problems may disproportionately impact *Satisfaction* and *Ability* in middle-aged adults due to increased time and cognitive demands by family and work responsibilities. This results in diminished sleep duration and greater functional impairment compared to older and frequently retired individuals [59], where total sleeping time was not associated with cognitive performance [60]. These findings highlight that depressiveness, anxiety, and sleep quality should be assessed and considered along with participants’ age, even when clinical cutoffs are not met, and advocate for a multifactorial approach toward metamemory.

It is important to note that the multifactorial approach of the MMQ also has drawbacks, including extended assessment time and the lack of an integrated single metamemory score. A limiting factor in our study is the online assessment lacking an objective marker of cognitive impairment. Despite a rigorous exclusion process to mitigate their impact, participants with cognitive impairment may remain in the sample. Ongoing studies will scrutinize the German MMQ’s onsite validity and assess its sensitivity in different pathologies and predictive value for patients’ quality of life. Furthermore, participants with lower educational background and with an age above 82 years are underrepresented in the current normative sample, warranting future studies with geriatric participants. Lastly, longer retest intervals, while common for follow-up visits, may have introduced bias, since not all participants could be reassessed.

Although subjective judgment about one’s own memory ability has repeatedly been shown to have small or no associations with objectified memory performance in standardized tests (e.g., Burmester et al. [61]), metamemory offers a distinct advantage over standardized memory assessments through its reflection of individual challenges [17]. Furthermore, it is sensitive to declines in personal performance, even in high-performing and mildly affected patients, where comparison to normative data might fall short. Consequently, the MMQ holds considerable value for clinical diagnosis and cognitive rehabilitation, where the goal is to regain adequate functionality within an individual’s environment [52, 62].

Taken together, our study indicates that the German MMQ scales show normal distribution, are valid and reliable, and provides normative data that can be useful to detect subjective memory impairment and monitor metamemory. Specifically, the MMQ scales *Satisfaction* and *Ability* sensitively reflect individual everyday memory problems, while the *Strategy* scale can be used to plan and monitor strategy applications and promote functional adaptation, for instance in cognitive interventions. The MMQ scales can be applied separately and easily integrated into clinical and research settings, particularly by employing a tablet or computerized version with automated scoring. In addition, the reliable change scores provide helpful measures for follow-up and therapy evaluation. This way, the German MMQ provides a sensitive assessment of metamemory as a personalized measures of functionality in acute care hospital, rehabilitation settings, observational studies, and clinical trials.

### Electronic supplementary material

Below is the link to the electronic supplementary material.

Supplementary file1 (XLSX 29 KB)

## Data Availability

The analysis scripts used in this study are available on the Open Science Framework at https://osf.io/x6e8f and the associated data is available on the open access repository of the Humboldt-Universität zu Berlin at https://doi.org/10.18452/26821.

## Appendix

See Fig. 6, Tables 5 and 6.

**Table 5:** Interpretation of percentiles

Percentile (range)	*z* (range)	Interpretation	*Satisfaction*	*Ability*	*Strategy*		
					*Total*	*Internal*	*External*
≥97.7	≥2	Very high	≥71	≥76	≥48	≥25	≥26
93.3–97.6	1.5 *to* 1.9	High	70	73–75	43–47	21–24	24–25
84.1–93.2	1 *to* 1.4	Above average	66–69	69–72	36–42	17–20	22–23
16.0–84.0	− 0.9 *to* 0.9	Average	43–65	49–68	17–35	5–16	11–21
6.8–15.9	− 1.4 *to* − 1.0	Below average	33–42	42–48	12–16	3–4	9–10
2.4–6.7	− 1.9 *to* − 1.5	Low	27–32	37–41	8–11	1–2	6–8
≤2.3	≤− 2	Very low	≤26	≤36	≤7	0	≤5

**Table 6:** Pearson correlations of MMQ scales with questionnaire data

	*Satisfaction*				*Ability*				*Strategy*			
	*r*	95% CI	*t*(334)	*p*	*r*	95% CI	*t*(334)	*p*	*r*	95% CI	*t*(334)	*p*
CPI-memory	− 0.67	[− 0.72, − 0.60]	− 16.34	<0.001	− 0.75	[− 0.80, − 0.70]	− 20.88	<0.001	0.42	[0.33, 0.50]	8.43	<0.001
CPI-attention	− 0.70	[− 0.75, − 0.64]	− 18.00	<0.001	− 0.74	[− 0.78, − 0.68]	− 19.93	<0.001	0.42	[0.32, 0.50]	8.34	<0.001
CPI-executive	− 0.62	[− 0.68, − 0.55]	− 14.46	<0.001	− 0.65	[− 0.71, − 0.59]	− 15.71	<0.001	0.40	[0.31, 0.49]	8.03	<0.001
GDS	− 0.53	[− 0.61, − 0.45]	− 11.50	<0.001	− 0.36	[− 0.45, − 0.27]	− 7.14	<0.001	0.25	[0.14, 0.34]	4.62	<0.001
BAI	− 0.41	[− 0.49, − 0.32]	− 8.18	<0.001	− 0.39	[− 0.47, − 0.29]	− 7.62	<0.001	0.27	[0.17, 0.37]	5.12	<0.001
PSQI	− 0.44	[− 0.53, − 0.35]	− 9.03	<0.001	− 0.40	[− 0.49, − 0.31]	− 8.04	<0.001	0.21	[0.10, 0.31]	3.83	<0.001
SF-12, mental health	0.36	[0.27, 0.45]	7.09	<0.001	0.30	[0.20, 0.39]	5.70	<0.001	− 0.18	[− 0.28, − 0.07]	− 3.35	0.001
SF-12, physical health	− 0.06	[− 0.17, 0.04]	− 1.18	0.239	− 0.05	[− 0.15, 0.06]	− 0.86	0.392	− 0.02	[− 0.13, 0.08]	− 0.44	0.659
